# Supplementary material for: Reduced competence to arboviruses following the sustainable invasion of Wolbachia into native Aedes aegypti from Southeastern Brazil
Source: Sci Rep. 2021 May 11;11:10039. doi: 10.1038/s41598-021-89409-8 (PMC8113270; doi:10.1038/s41598-021-89409-8)
Supplement: Supplementary file 1 — Supplementary Information [file 41598_2021_89409_MOESM1_ESM.docx]

# **Supplementary Information**

**Reduced competence to arboviruses following the sustainable invasion of *Wolbachia* into native *Aedes aegypti* from Southeastern Brazil**

**João Silveira Moledo Gesto^1,3+^, Gabriel Sylvestre Ribeiro^1,3+^, Marcele Neves Rocha^1,3+^, Fernando Braga Stehling Dias^2,3^, Julia Peixoto^3^, Fabiano Duarte Carvalho^1^, Thiago Nunes Pereira^1^, Luciano Andrade Moreira^1,3*^**

^1^ Grupo Mosquitos Vetores: Endossimbiontes e Interação Patógeno Vetor, Instituto René Rachou, Fiocruz Minas, Belo Horizonte, MG, Brazil.

^2^ Gabinete da Presidência, Fiocruz, Rio de Janeiro, RJ, Brazil.

^3^ World Mosquito Program, Fiocruz, Rio de Janeiro, RJ, Brazil.

**Supplementary Figure S1. Map of egg-release sites.** Spatial distribution of Mosquito Release Containers (MRCs) (blue circles) across Jurujuba’s sectors. At 15-days intervals, each MRC was loaded with a fresh batch of *w*Mel-infected eggs. Maps were created with ArcGIS Desktop 10.7 (Esri Inc., <https://www.esri.com/en-us/arcgis/products/arcgis-desktop/overview>) using OpenStreetMap source codes (OpenStreetMap Contributors), under the license CC-BY-SA 2.0.


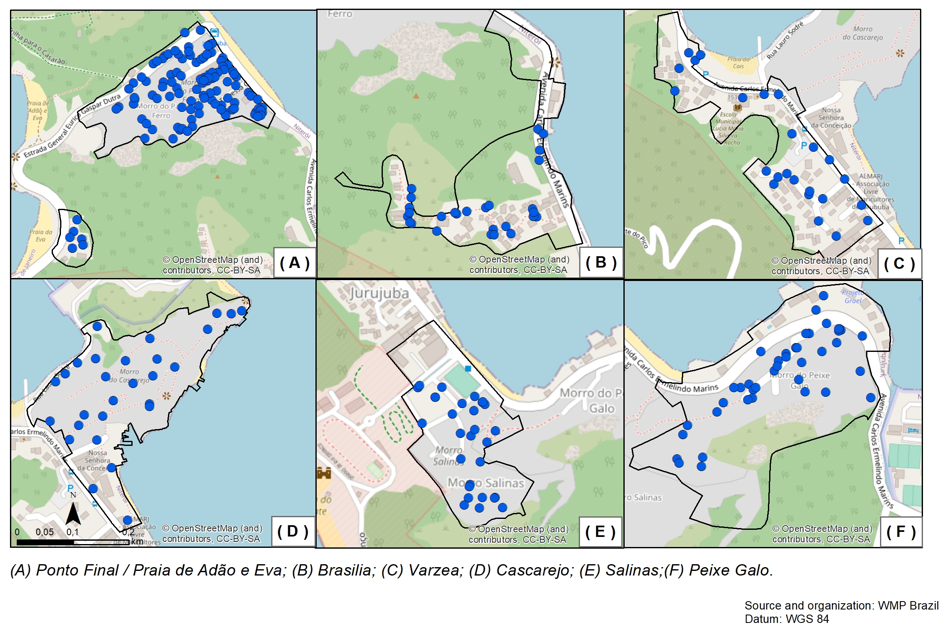


**Supplementary Figure S2. Map of monitoring sites.** Spatial distribution of BG-sentinel traps (black circles) across Jurujuba’s sectors. Traps were monitored weekly to assess *Wolbachia* frequency in field specimens. Maps were made with ArcGIS Desktop 10.7 (Esri Inc., <https://www.esri.com/en-us/arcgis/products/arcgis-desktop/overview>) using OpenStreetMap source codes (OpenStreetMap Contributors), under the license CC-BY-SA 2.0.


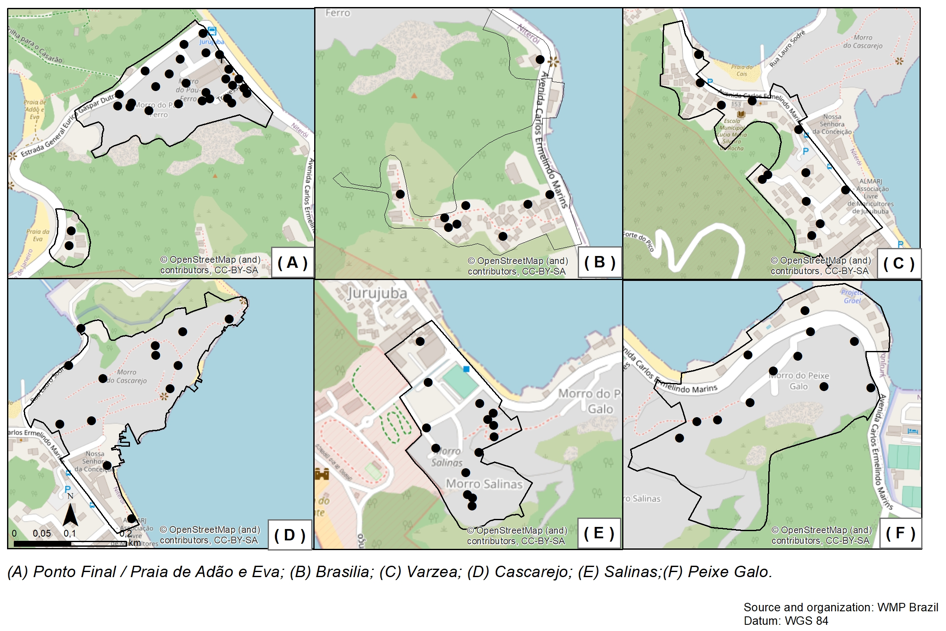


**Supplementary Figure S3. *Wolbachia* prevalence and density in virus-challenged samples.** *Wolbachia* frequency and whole-body density was assessed in Urca (grey) and Jurujuba samples (green) orally-challenged with ZIKV and DENV. Whole-body density is a relative quantity, expressed as the ratio between *Wolbachia*-specific WD0513 and endogenous RPS17 genetic markers. Violin plots depict the distribution of density data from Urca and Jurujuba samples, with the proportion of *Wolbachia*^+^ individuals shown as fractions underneath. *Wolbachia*^–^ Jurujuba individuals were flagged by empty dots. Medians are shown in solid and quartiles in dashed red lines. Graph were created with GraphPad Prism 8 (https://www.graphpad.com).


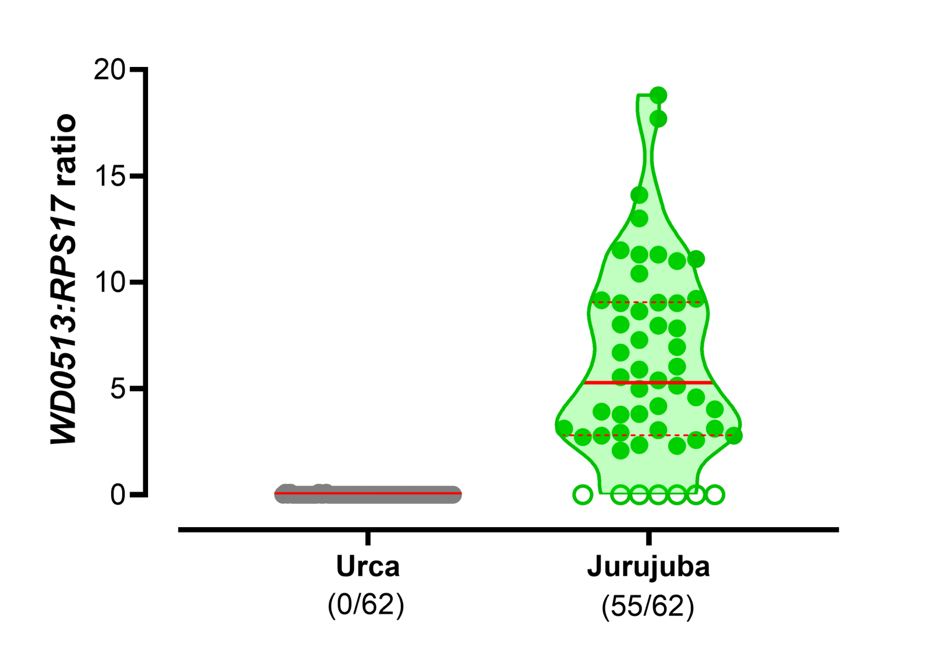


**Supplementary Table S1. Egg release schedules in Jurujuba.** Along the *Wolbachia* field deployment period, Mosquito Release Containers (MRCs) were set up across Jurujuba’s territory in a variable fashion for each of its sectors. The release schedules, MRCs allocation and the mean number of released eggs (per MRC unit) are listed.

| **Sector** | **MRCs** | | | |  | **Release Schedule** | | |
| --- | --- | --- | --- | --- | --- | --- | --- | --- |
|  | Group A | Group B | Total | Eggs / Unit  (mean + SD) |  | Start | End | Length (weeks) |
| Ponto Final | 57 | 58 | 115 | 176 ± 48 |  | 25/08/2015 | 16/02/2016 | 25 |
| Cascarejo | 28 | 28 | 56 | 209 ± 51 |  | 07/06/2016 | 10/01/2017 | 31 |
| Brasília | 14 | 14 | 28 | 260 ± 38 |  | 13/09/2016 | 29/11/2016 | 11 |
| Salinas | 30 | 30 | 60 | 258 ± 40 |  | 13/09/2016 | 03/01/2017 | 16 |
| Varzea | 12 | 12 | 24 | 271 ± 44 |  | 13/09/2016 | 08/11/2016 | 8 |
| Peixe Galo | 21 | 21 | 42 | 270 ± 19 |  | 20/09/2016 | 03/01/2017 | 15 |
| Praia de Adão e Eva | 6 | - | 6 | 258 ± 35 |  | 20/09/2016 | 11/01/2017 | 16 |

**Supplementary Table S2. BG-Sentinel traps allocation in Jurujuba.** Number of BG-Sentinel traps allocated per Jurujuba sector, since the beginning of *Wolbachia* field monitoring until recent days. Traps were widely used and reached their maximum numbers at deployment periods, when monitoring with the best possible spatial resolution was critical, and then partially demobilized into the post-release phase following a successful *Wolbachia* invasion (ie. stable high-frequency infection indexes). Revision of schedules and trap numbers was necessary to allow a viable long-term monitoring activity across Jurujuba’s territory.

| **Jurujuba’s sector** | **Number of BG-traps** | |  | **Monitoring schedule** | |
| --- | --- | --- | --- | --- | --- |
|  | Max. | Min. |  | Start | End |
| Ponto Final | 26 | 3 |  | 08/09/2015 | 30/12/2019 |
| Cascarejo | 13 | 2 |  | 21/06/2016 | 30/12/2019 |
| Brasília | 9 | 2 |  | 27/09/2016 | 30/12/2019 |
| Salinas | 14 | 4 |  | 27/09/2016 | 11/12/2018 |
| Varzea | 11 | 2 |  | 27/09/2016 | 30/12/2019 |
| Peixe Galo | 12 | 1 |  | 27/09/2016 | 11/12/2018 |
| Praia de Adão e Eva | 2 | 2 |  | 06/10/2016 | 14/02/2017 |

**Supplementary Table S3. qPCR primes and probes.** List of primers and probes for the molecular detection and quantification of ZIKV, DENV and *Wolbachia*.

| **PRIMER** | **NUCLEOTIDE SEQUENCE (5’ 🡪 3’)** |
| --- | --- |
| *ZIKV* | |
| ZIKV 835Forward | TTGGTCATGATACTGCTGATTGC |
| ZIKV 911Reverse | CCTTCCACAAAGTCCCTATTGC |
| ZIKV Probe | **FAM**/CGGCATACA/**ZEN**/GCATCAGGTGCATAGGAG/**3IABkFQ** |
| *DENV* | |
| DENV Forward | AAGGACTAGAGGTTAGAGGAGACCC |
| DENV Reverse | CGTTCTGTGCCTGGAATGATG |
| DENV Probe | **TEX615**/AACAGCATATTGACGCTGGGAGAGACCAGA/**3IAbRQSp** |
| *Wolbachia* | |
| WSPTM2 Forward | CATTGGTGTTGGTGTTGGTG |
| WSPTM2 Reverse | ACACCAGCTTTTACTTGACCAG |
| WSPTM2 Probe**^a^** | **FAM**/TCCTTTGGA/**ZEN**/ACCCGCTGTGAATGA/**3lAbRQSp** |
| WSPTM2 Probe**^b^** | **CY5**/TCCTTTGGA/**TAO**/ACCCGCTGTGAATGA/**3lAbRQSp** |
| *Ae. aegypti* RPS17 | |
| RPS17S Forward | TCCGTGGTATCTCCATCAAGCT |
| RPS17S Reverse | CACTTCCGGCACGTAGTTGTC |
| RPS17S Probe | **HEX**/CAGGAGGAG/**ZEN**/GAACGTGAGCGCAG/**3lABkFQ** |

^a^ Probe used for *Wolbachia* field monitoring and density level assessment

^b^ Probe used for viral diagnosis

**Supplementary Datasheet S1.** Monitoring data of total *Ae. aegypti* caught by BG-Sentinel traps in Jurujuba. Each sector is represented in a separate sheet. Pre-release data is limited to Ponto Final.
